# Immune Responses to Inhalant Mammalian Allergens

**DOI:** 10.3389/fimmu.2014.00234

**Published:** 2014-05-21

**Authors:** François Hentges, Cathy Léonard, Karthik Arumugam, Christiane Hilger

**Affiliations:** ^1^Laboratory of Immunogenetics and Allergology, CRP-Santé, Luxembourg, Luxembourg; ^2^Laboratory of Retrovirology, CRP-Santé, Luxembourg, Luxembourg

**Keywords:** secretoglobin, lipocalin, albumin, allergen, cellular response, IgE response, cross-reactivity

## Abstract

In Europe and the USA, at least one person in four is exposed every day to inhalant allergens of mammalian origin, a considerable number is regularly exposed for professional reasons and almost everyone is occasionally exposed to inhalant allergens from pets or domestic animals. The production of IgE to these inhalant allergens, often complicated by asthma and rhinitis, defines the atopic status. However, the immune response to these allergens largely imprints the cellular immune compartment and also drives non-IgE humoral immune responses in the allergic and non-allergic population. During the recent years, it has become clear that IgE antibodies recognize mammalian allergens that belong to three protein or glycoprotein families: the secretoglobins, the lipocalins, and the serum albumins. In this article, we review the humoral and cellular immune responses to the major members of these families and try to define common characteristics and also distinctive features.

## Introduction

The immune system of the respiratory tract of children and adults is continuously being exposed to inhaled particles of inorganic and organic origin. Some particles or molecules have potential adjuvant activities, some have allergenic potential such as mite allergens, other molecules for instance of human origin are devoid of immunogenic or allergenic properties. In their daily environment, most people are also exposed to allergens of mammalian origin. The exposure rate can be high for people having pets at home, for farmers who keep or raise domestic animals, and for persons who have other types of professional contact with animals, for instance veterinarians or animal workers in a laboratory setting. However, even people that do not have direct contact with animals may have contact with inhalant allergens that have been shed by animals or that have been carried to public places by animal owners, as documented by the presence of cat allergens in schools and other public places (1). Indeed, allergic sensitization to animal allergens is common in persons who do not have pet animals at home. Data from a large pan-European study show that about 27% of the patients referred to an allergy center for allergic reactions to inhalant allergens were sensitized to cat and/or dog (2). Sensitization was particularly high in Nordic countries, the highest sensitization rate for dogs reaching 56% in Denmark.

As dendritic cells combine antigen presentation capacities with sensing of signals of innate immunity, they are the master players in all types of adaptive immunity. The important role of airway epithelial cells in alarming dendritic cells for allergic sensitization has more recently been highlighted (3, 4). In contrast to certain other inhalant allergens (Der p 1, Der p 2), an inherent capacity of mammalian allergens to trigger the pathways of innate immunity has not been convincingly shown, although a recent publication argues for an enhancing activity of some mammalian allergens on toll-like receptor (TLR) activation by lipid ligands (5). Allergens of mammalian origin do not only induce IgE antibodies but also IgG isotypes and different T cell responses (Th2, Th1, Th17, and regulatory T cells) in allergic and non-allergic persons. We will review the immune responses to three major mammalian allergen families: secretoglobins, lipocalins, and serum albumins. We will focus on the most prominent of their members and try to distinguish common and specific characteristics based on published data.

## Secretoglobins

**Figure 1:**
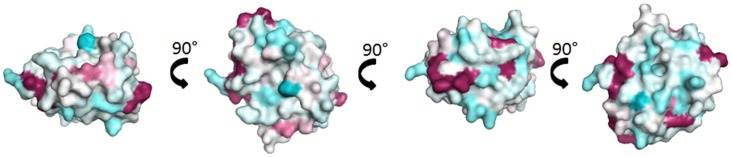
**Comparison of Fel d 1 and Ory c 3 composing the secretoglobin group**. Surface representation of Fel d 1 (2EJN) (7) colored by sequence conservation with Ory c 3 (Q9GK63; Q9GK67) (9). Sequence conservation was determined through Consurf (10), the highly variable sequence conservation was identified as deep blue, the average was in white, and the conserved sequences are denoted in deep red.

Fel d 1, the major cat allergen, is a 35-kDa tetrameric glycoprotein formed of two non-covalently linked heterodimers (6, 7). Each heterodimer comprises a light alpha-chain (or chain 1) and a heavy beta-chain (or chain 2) containing an *N*-linked oligosaccharide (8). Until the recent description of rabbit lipophilin Ory c 3 (9), Fel d 1 was the only known allergen of the secretoglobin family. Both molecules display little sequence identity (24%) despite a high structural identity. There are no protein stretches of more than three consecutive identical amino acids which are common to the two molecules and which could form identical linear epitopes. Surface representation of Fel d 1 overlaid with the sequence of Ory c 3 does not show evidence for significant common discontinuous epitopes (Figure 1). Indeed, IgE cross-reactivity between Fel d 1 and Ory c 3 could not be shown (9).

### Exposure and effect on the humoral immune response

Cats are present in 24% of European Union and up to 37% of United States households (11). The level of airborne Fel d 1 in homes with a cat was found to be in the range of 1.8–578 ng/m^3^, a comparable level of airborne Fel d 1 ranging from 2.8 to 88.5 ng/m^3^ was also measured in 25% of homes without a cat (12). The quantity of Fel d 1 inhaled on airborne particles by children in homes with a cat has been estimated to be 1 μg/day, which is about 100 times the quantity of mite or pollen allergens inhaled in 1 day (13, 14). At the age of 4, already 5.8% of children of a U.K. birth cohort study were sensitized to cat (15) while 16.9% of adults were sensitized in another study (16). In a recent study including 96 cat-allergic patients, IgE antibodies to Fel d 1 represent on average 55% of a cat-specific IgE response, however, with a range from 0 to 100%. Fel d 1 was the cat allergen that bound the highest amount of IgE in 65% of the patients (17). Surprisingly, children exposed to cat during the first year of life are less often skin prick test positive to cat at 12–13 years than controls (18). Exposure to high concentrations of Fel d 1 was found to be associated with lower sensitization in terms of specific IgE but increased levels of IgG (and IgG4) antibodies to Fel d 1 which has led to the concept of “modified Th2 response” (19). Exposure to cat and Fel d 1 was associated with three patterns of humoral responses: an allergic response characterized by the presence of IgE and IgG (often IgG4) to Fel d 1, a modified Th2 response characterized by presence of IgG (mostly IgG4) antibodies but absence of IgE response to Fel d 1, and thirdly an absence of humoral response to Fel d 1 characterized by failure to produce specific IgE or IgG to Fel d 1, even though exposure to Fel d 1 took place (20). However, the analysis of IgE, IgG, and IgG4 antibodies to Fel d 1 in relation to wheezing in two large birth cohorts showed that allergen-specific IgG but not IgG4 antibody levels were related to improved wheezing in children with Fel d 1-specific IgE (21). At this point, it might be interesting to note that a phase I/II a clinical trial using intralymphatic immunotherapy with a technology called modular antigen translocation (MAT) linking Fel d 1 to a construct enhancing its presentation by the MHC class II pathway, increased Fel d 1-specific IgG4 production (22). Thus, exposure to high concentrations of Fel d 1 in the daily environment is associated with clinical improvement in parallel to increased levels of Fel d 1-specific IgG and in particular IgG4 antibodies (19). A similar modified Th2 response can be achieved by high Fel d 1 loading of the antigen-presenting pathway by intralymphatic immunotherapy and MAT technology (22). This is in line with the concept that a modified Th2 response is associated with high antigen exposure.

### T cell response as measured *ex vivo*

The frequency of Fel d 1-peptide-specific T cells in peripheral blood as assessed by tetramer technology using a chain 1 peptide (aa 32–48) – DRB1*01:01 tetramer complex in a population of HLA – DRB1*01:01 positive cat-sensitized patients with atopic dermatitis and controls gave the following results. Atopic dermatitis patients had 3–53:100.000 and controls about 3:100.000 tetramer-positive CD4 T cells in their peripheral blood (*ex vivo*). The majority of about 80% expressed a central memory phenotype (with high surface expression of CCR7, CD62L, CD27, and CD28) (23). Comparable results were obtained for Fel d 1-specific CD4 cells with tetramers containing six different peptides bound to six different HLA class II molecules (24). Peptide-specific CD4 cells ranged from 1:7000 to 1:300000 in allergic subjects. In subjects without allergy, tetramer-positive CD4 T cells were barely detectable. Nearly all cells exhibited a central memory phenotype, however, CCR7 expression was heterogeneous. A relevant percentage of cells were CCR4^+^, interpreted as a commitment to migrate to non-lymphoid sites. In comparison, in birch-allergic persons, the percentage of CD4 T cells recognizing MHC class II tetramers containing an immunodominant peptide was about 500:100.000 in allergic and 300:100.000 in non-allergic persons during the peak pollen season (25). This is more than one order of magnitude higher than for Fel d 1 in cat-allergic persons. In birch-allergic persons, the cells were mainly of an effector memory phenotype (IL-5 and some IL-10). The cells of non-allergic persons were of central memory phenotype (secreting IFN-γ and IL-10) in response to the allergen. Peptide-positive cells could not be detected directly *ex vivo* outside the pollen season. Their number was estimated to be at that time point 2–3 logs lower than during the peak pollen season (25). One possible explanation for this difference in peptide-specific T cells could be a smaller initial peripheral T cell repertoire against Fel d 1 due to a stronger thymic deletion of T cells recognizing epitopes of mammalian origin (phylogenetically closer to human epitopes) than T cell recognizing epitopes on molecules of plant origin. It is, however, more likely that the greater number of CD4 T cells recognizing MHC class II tetramers loaded with immunodominant pollen-derived peptides is due to the seasonal boosting of cellular immune response linked to pollen exposure.

### Peptides and peptide immunotherapy

By T cell epitope mapping with peptides, amino acid positions 1–10 and 16–24 of Fel d 1 chain 2 were found to be associated with a HLA-DR7-restricted secretion of high IL-10, respectively IFN-γ in PBMC cultures of persons with a modified Th2 response (20). Previous work had defined Fel d 1 T cell epitopes mainly on chain 1, by means of cell proliferation assays of T cell lines established from persons allergic to cats (26). Intradermal administration of short overlapping peptides derived from chain 1 of Fel d 1 that did not cross-link IgE, did not elicit a visible early or late cutaneous response, but caused late asthmatic reactions in 9/40 cat-allergic asthmatics (27). The individual peptides were able to induce proliferation and IL-5 secretion in a HLA class II restricted manner from T cell lines established from asthmatic subjects, indicating IgE-independent, T cell-dependent allergic reaction. Determination of the binding affinities of Fel d 1 peptides to 10 commonly expressed HLA-DR molecules, combined with their proliferative and cytokine responses (IFN-γ, IL-10, and IL-13) in cat-allergic persons allowed a comprehensive identification of immune-dominant sequences including those on chain 2 (28). A short peptide immunotherapy course with a combination of promiscuous peptides (serving as restriction element to different HLA-DR molecules) improved the ocular and nasal components of rhino-conjunctivitis symptoms in subjects with cat allergy, with a treatment effect persisting 1 year after the start of treatment (29). This approach uses only short peptides (12–16 amino acids long) which are not recognized by IgE antibodies able to trigger an early asthmatic response through mediator release by basophils and mast cells. These short peptides are also not likely to be recognized by surface-bound IgM and thus interfere at the immature B cell level. Their effect is rather due to a dampening of the effector T cells for instance IL-5-secreting T cells implicated in the late allergic asthmatic response. The impact could be due to changes of the helper or regulatory cellular functions. Of course, an immune system modified by this approach at the cellular level could secondarily be susceptible to changes at the humoral level after later inhalation of Fel d 1 molecules present in the environment.

## Lipocalins

**Figure 2:**
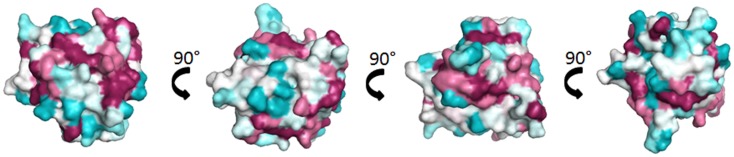
**Comparison of the cross-reactive lipocalin group from horse (Equ c 1), cat (Fel d 4), and dog (Can f 6)**. Surface representation of Equ c 1 (1EW3) (69) colored by sequence conservation with Fel d 4 (Q5VFH6) (70), Can f 6 (H2B3G5) (35).

**Figure 3:**
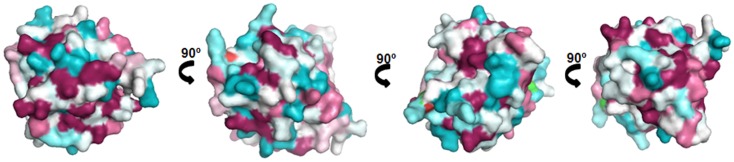
**Comparison of the cross-reactive lipocalin group from rat (Rat n 1), mouse (Mus m 1), and rabbit (Ory c 4)**. Surface representation of Rat n 1 (1MUP) (71) colored by sequence conservation with Ory c 4 (U6C8D6) (72) and Mus m 1 (P02762) (73).

Lipocalins represent the largest group of mammalian inhalant allergens. They are major allergens from dog, horse, cattle, guinea pig, rat, mouse, rabbit, and hamster (30). Lipocalins have a common tertiary structure composed of a central β-barrel formed of eight anti-parallel β-strands (31). Lipocalins were shown to carry small hydrophobic molecules such as retinol, steroids, odorants, and pheromones in their internal binding pocket. Despite a highly conserved structural similarity, lipocalins generally have a very low amino acid identity, which for some of them can be lower than 20% (32), a fact that makes IgE cross-reactivity among these lipocalins unlikely. Until recently, it was assumed that IgE cross-reactivity between lipocalins would be limited to isolated epitopes with great amino acid identity between lipocalins (33). However, besides lipocalins with very low amino acid identity, a group with greater homologies and IgE cross-reactivity has been individualized. It comprises the following allergens: Fel d 4 (cat), Can f 6 (dog), Equ c 1 (horse), Ory c 4 (rabbit), Mus m 1 (mouse), and Rat n 1 (rat) (Table 1). Pairwise sequence comparisons show identities in the range of 47–67% whereas other cat and dog lipocalins have only weak identities. There is also another lipocalin pair namely Fel d 7 and Can f 1 that shares 63% identity at the amino acid level and which might give rise to cross-sensitization. Concerning the main group, IgE cross-reactivity was first shown between mouse and rat urinary lipocalins using sIgE inhibition (34). Indeed at the amino acid level, there exists an identity of 64% between the major rat lipocalin Rat n 1 and mouse lipocalin Mus m 1. More recently considerable IgE cross-reactivity was shown between dog lipocalin Can f 6 and cat Fel d 4 (amino acid identity 67%) and between these lipocalins and horse lipocalin Equ c 1 (35–37). IgE cross-reactivity between Equ c 1 and Can f 6 was shown to be clinically relevant in a horse- and dog-allergic patient who showed no specific IgE to known dog allergens except Can f 6 (36). IgE reactivity of this patient to Can f 6 could be completely inhibited by Equ c 1. Can f 6 and Equ c 1 share 57% amino acid identity. Table 1 gives a summary of amino acid identity and cross-reactivity between members of the cross-reactive lipocalin group. Structural identity between Equ c 1, Fel d 4, and Can f 6 are visualized in Figure 2, structural identities between Mus m 1, Rat n 1, and Ory c 4 are shown in Figure 3.

**Table 1:** **Amino acid identities (%) and IgE cross-reactivity between members of a mammalian lipocalin subgroup**.

Fel d 4		67 (35, 37)^a^	67 (37)^a^	63	49	55
Can f 6	67 (35, 37)^a^		57 (37)^a^	58	47	52
Equ c 1	67 (37)^a^	57 (36)^b^ (37)^a^		52	46 (33)^a^	47
Ory c 4	63	58	52		51	54
Mus m 1	49	47	46	51		64 (34)^a^
Rat n 1	55	52	47	54	64 (34)^a^	
	Fel d 4	Can f 6	Equ c 1	Ory c 4	Mus m 1	Rat n 1

*Amino acid identities between lipocalins are given as %*.

*^a^Documented IgE cross-reactivity (allergen-specific inhibition)*.

*^b^Documented IgE cross-reactivity with clinical history of cross-reactivity*.

*Literature references are given in brackets*.

*Vertical: sensitizing allergen. Horizontal: cross-reactive allergen*.

### Exposure and effect on the humoral immune response

Lipocalins are shed into the environment by animal dander and secretions. Dog allergen Can f 1 is ubiquitously present in human residential environment. The most probable mechanism of allergen transfer to public places is clothing (1). Lipocalins are found both in airborne and settled dust. Although Fel d 1 and Can f 1 belong to different protein families, a number of studies have shown that allergen levels found in airborne or settled dust are in the same range of magnitude for both molecules (38).

The importance of mouse allergens was initially demonstrated in the occupational setting (39). However, the role of mouse allergen exposure in domestic environments has also gained attention. Mus m 1, the major mouse allergen, is prevalent in US urban and suburban residential environments. It has been shown to be related to asthma morbidity (40–42). Importantly, airborne and settled dust mouse allergen levels were shown to vary over time in a given home, implying that environmental conditions of sensitized patients may change from high to low exposure and vice versa. Conditions of high exposure reached values up to 5.68 ng/m^3^, which are comparable to measurements obtained in animal facilities.

The effect of allergen concentration on the immune response has been addressed in a prospective study analyzing the immune response of newly hired employes of a mouse facility over time. The concentrations of Mus m 1 in the air ranged from 0.09 to 9.88 ng/m^3^, the median of the allergen concentrations over time being 0.69 ng/m^3^. By 24 months, 23% of the participants had developed a positive SPT. Interestingly, the risk of becoming positive was not linear, increasing from low to moderate levels of exposure, peaking at approximately 1.2 ng/m^3^, and then decreasing from moderate to high levels of exposure (43). Eight percent had developed mouse-specific IgG4, the incidence increasing with increasing levels of mouse allergen exposure. Ten percent of the participants had developed mouse-specific IgG1–3 with a non-significant association with higher exposure. A previous cross-sectional occupational study had shown that high exposure to rats was associated with lower rates of symptoms and specific IgE to rat urine allergen (containing Rat n 1) but an increased frequency of highly specific IgG and IgG4 (44).

A recent study addressing specific IgE, IgG1, IgG4 levels and the peripheral blood mononuclear cytokine responses to eight separate cat allergens in cat-allergic and in cat non-allergic persons showed IgG4 antibodies to Fel d 4 in 12% of allergic persons and in only 3% of non-allergic persons (17). Although IgG1 antibodies to Fel d 1 were found in allergic and non-allergic persons, there were none detected against Fel d 4. Another recent study analyzing on a microarray system of IgE and IgG antibodies to a series of dog, cat, and horse allergens showed that almost all the patients but also controls had IgG antibodies to the cross-reacting lipocalin group (Fel d 4, Can f 6, and Equ c 1) (45).

### T cell responses to lipocalin allergens

Rat n 1 allergen purified from rat urine and pools of overlapping peptides spanning Rat n 1 were tested for the proliferative responses of PBMCs of rat-allergic individuals, rat-exposed but non-allergic individuals and non-exposed, non-allergic individuals (46). The proliferative responses to Rat n 1 of the three groups were similar and weak with a median stimulation index below 2 (but with an extremely great range between 0.01 and 22.2). Nevertheless, four peptide pools induced with high frequency weak positive responses in allergic individuals in comparison to non-allergic referents (46). Interestingly, the levels of IL-5 were significantly increased in supernatants of PBMCs stimulated with rat urinary protein from allergic subjects, compared to non-allergic control subjects and even more so when compared to non-exposed controls when stimulated with rat urinary protein. Four similar epitope areas had previously been defined in the cow dander allergen Bos d 2 in cow-asthmatic individuals (47). Here also, the proliferative response of PBMCs to native Bos d 2 had been weak and the four epitopes concentrated on the conserved regions of the molecule had been most clearly defined by the proliferative response of a number of T cell clones established from five cow-allergic patients. According to their cytokine-producing pattern, 37% of the clones were classified as Th0-like (IL-5, IL-4, IFN-γ), 7.9% were Th1-like (IFN-γ), and 55% were Th2-like (IL-4, IL-5). Using HLA class II-peptide tetramer technology, naïve Bos d 2-specific T cells of PBMC cultures of individuals with or without allergy were of similar frequency, whereas the frequency of CD4^+^, CD45RO^+^ memory cells appeared to be higher in subjects with allergy (48). These findings were confirmed by a recent study on the CD4 T cell response to Equ c 1, the major horse lipocalin allergen (49). Allergic and non-allergic subjects had a similar low frequency of Equ c 1-specific CD4 T cells, but the cells from the allergic subjects had a stronger proliferative response, were predominantly Th2 biased and originated mostly from memory CD4 T cells.

T cell epitopes have also been mapped for the major allergens of dog and horse, Can f 1 and Equ c 1, respectively (50, 51). Seven epitope regions were defined for Can f 1 and on average, patients recognized three epitopes. T cell lines from allergic patients produced more IL-4 than those from healthy controls. However, depending on the peptide used, they produced also more IL-10 or more IFN-γ (52). A comparison of Can f 1-specific T cell lines generated from dog-allergic and non-allergic but exposed persons showed an absence of IL-4 secreting T cell lines in non-allergic persons, while IL-5, IL-10, IFN-γ, and IL-17 lines were found in both groups although at different frequencies (53). Specific T cell lines established from 10 horse-allergic patients determined 8 epitope regions and 1 dominant epitope in the C-terminal region of Equ c 1 (51). Similarly to the findings on Rat n 1 and Bos d 2, epitopes are clustered in a few regions and they elicit only weak T cell responses in PBMCs which are enhanced in the T cell lines obtained after several stimulation cycles (51).

## Serum Albumins

Serum albumins represent the major protein component in the circulatory system of mammals. They are produced by hepatocytes and have a molecular weight in the range of 66–69 kDa. They contribute significantly to colloid osmotic blood pressure and aid in the transport of many endogenous and exogenous ligands. The albumin molecule is very flexible, it has an α-helical structure stabilized by several disulfide bridges and is divided into three domains (54). Serum albumins are also present in body fluids and on dander. In house dust samples, concentrations of human serum albumin (HSA) have been measured in the range of 40–301 μg/g dust (55). Again, this is the range of the settled dust amounts measured for Fel d 1 and Can f 1, two molecules representative of a different allergen family. There are no commercial tools available up to date for the specific measurement of animal albumins in dust samples.

### Immune response to serum albumins

The immune response to serum albumins is well-documented at the antibody level, however, there are few data on the cellular response to serum albumins. Specific IgE to dog serum albumin (DSA) were first described in dog dander asthmatic children who were prick test positive to DSA (9 out of 80) (56). The titers of anti-DSA IgG measured did not correlate with specific IgE titers. Lymphocyte transformation tests in anti-DSA IgE-positive patients were weak except for one patient. In subsequent reports, the importance of DSA as a cross-reactive allergen was established. In a study with 110 dog-allergic patients, 35% were shown to have IgE against DSA (57). IgE antibodies from several selected patients bound also to albumins from other species such as cat, mouse, and rat. Histamine release with the different albumins was shown for one patient. A more extensive analysis of cross-reactivity was performed on a sample of 200 patients allergic to animal dander (58). Thirty percent of these patients presented IgE reactivity to albumins in animal hair/dander extracts and were further tested in dot-blot experiments for cross-reactivity with 11 different mammalian albumins. The majority of the patients’ IgE recognized a large spectrum of albumins; some, however, displayed a highly selective reactivity.

### IgE cross-reactivity between inhaled and ingested or systemically administered serum albumins

Despite their high level of cross-reactivity, albumins were considered minor allergens without documented clinical significance. This was challenged by a case report of a severe anaphylactic reaction after artificial insemination in a patient sensitized to animal dander (59). Bovine serum albumin (BSA), a compound of the medium used, could be identified as trigger for the reaction. A first report on cat-allergic patients experiencing anaphylactic reactions upon consumption of pork meat coined the term pork–cat syndrome (60). Another study investigated the role of serum albumin in this syndrome. The sensitization of cat-allergic patients to cat serum albumin was analyzed and possible cross-sensitization profiles to pork albumin were determined by inhibition assays (61). The frequency of sensitization to cat albumin ranged between 14 and 23%, depending on the cohort, while sensitization to porcine serum albumin ranged from 3 to 10%, respectively. About 1/3 of the patients sensitized to porcine serum albumin are likely to experience adverse reaction by the consumption of pork, especially ham or sausages, as albumins are heat-labile proteins. Statistically, about 1–3% of cat-allergic patients would be at risk for adverse reactions to pork (61). The described cases all originated from Europe, but recently several cases with immediate type allergic reactions upon pork consumption have been reported in the US (62). Testing for serum IgE to cat and pork serum albumin allows discriminating this syndrome from the reactions of delayed food allergy related to the presence of IgE directed to alpha-gal sugar determinants on meat (63). The high level of IgE cross-reactivity between serum albumins hampers the determination of the sensitizing molecule. The clinical history of sensitization, the level of specific anti-albumin IgE titers as well as IgE inhibition data have to be taken into account to establish a correct diagnosis. Although IgE cross-reactivity is most frequent between mammalian albumins, cross-reactivity may also occur between cat and chicken albumin which share only 46% identical amino acids (64). BSA is an important allergen of meat and milk. IgG and IgA responses to BSA and different fragments thereof have been analyzed in three cohorts: unselected persons, new-onset insulin-dependent diabetes mellitus patients, and atopic patients (65). IgG and IgA antibodies to BSA were inversely correlated with age in the normal population. In all three cohorts, IgG antibodies recognized all three BSA domains with an equivalent frequency, however, only 31–46% of the subjects’ IgA antibodies were able to bind to the N-terminal part of the BSA molecule. This finding correlated with the fact that the N-terminal domain was also the first to be degraded in simulated gastric fluid experiments, suggesting that systemic IgG antibody responses and gut-associated lymphoid tissue IgA responses to the food allergen are independent.

Mammalian serum albumins display a very high amino acid identity (72–82%) to HSA (66). This leaves very little space for the discrimination of self from non-self. It is interesting to note that up to now no clear autoimmune reaction to HSA has been proven. The role of the so-called ABBOS peptide, an epitope present on the BSA molecule, has been controversially discussed in the development of insulin-dependent diabetes mellitus (67).

It has been hypothesized that, at least for food allergens, molecules with a high degree of similarity to human homologs would be poorly immunogenic (68). Above a threshold of 62% sequence identity, proteins were found to be rarely allergenic. BSA, an allergen of cow’s milk is an exception as it shares 75.6% identity with HSA. The respiratory allergenic albumins present even identities of 81.7% (cat), 79.8% (dog), and 76.1% (horse) to HSA. The structural features of mammalian and avian serum albumins have been addressed in a recent review by Chruszcz et al. (66). Their three-dimensional structure is crucial for antibody binding and allergenicity. Albumins are sensitive to heat treatment and thoroughly cooked food is generally tolerated by allergic patients.

## Conclusion

Inhalant mammalian allergens are capable of eliciting a large variety of immune responses, of which production of specific IgE is only one. At the humoral level, a particular aspect is the high IgE cross-reactivity mainly within the serum albumins but also a cross-reactive lipocalin group. At the T cell level, the overall proliferative response to mammalian allergens is rather low, with nevertheless important variations. Allergen-specific CD4 T cells in peripheral blood of allergic persons are slightly more frequent than in non-allergic persons and predominantly have a Th2 central memory profile. The studies with Fel d 1 peptides have clearly established the existence of an effector cellular immune response, which has a pathogenic potential which is independent of the humoral IgE response. High allergen exposure, whether natural or immunotherapy-induced, is correlated to clinical benefit in parallel to the production of high titers of allergen-specific IgG4. A causal relationship between the two observations needs still to be proven. The issue of an autonomous immunogenicity and allergenicity of mammalian allergens is still elusive, the possibility of an allergenic bystander effect of other inhaled particles with adjuvant properties is a realistic option. At last, mammalian allergens, especially those whose amino acid sequence is close to that of their human homologs, are unique tools to study the immune response at the frontier between self and non-self.

## Conflict of Interest Statement

The authors declare that the research was conducted in the absence of any commercial or financial relationships that could be construed as a potential conflict of interest.
